# How to Preserve Fertility in Reproductive-Age Women with Cancer

**DOI:** 10.3390/jcm14061912

**Published:** 2025-03-12

**Authors:** Sébastien Jaeck, Chloé Depuydt, Valérie Bernard, Omar Ammar, Claude Hocké, Jennifer Carrière, Lucie Chansel-Debordeaux

**Affiliations:** 1 Reproductive Biology Unit-CECOS, University Hospital of Bordeaux, 33000 Bordeaux, France; sebastien.jaeck@chu-bordeaux.fr (S.J.); chloe.depuydt@chu-bordeaux.fr (C.D.); 2 U1312-BRIC Team Biotherapies Genetics and Oncology-BioGO, Bordeaux University, 33000 Bordeaux, France; valerie.bernard@chu-bordeaux.fr; 3 Gynecological Surgery, Medical Gynecology and Reproductive Medicine Department, University Hospital of Bordeaux, 33000 Bordeaux, France; claude.hocke@chu-bordeaux.fr (C.H.); jennifer.carriere@chu-bordeaux.fr (J.C.); 4 Clinical Research and Development Division, Louise, 33300 Bordeaux, France; omar@louise.life

**Keywords:** fertility preservation, chemotherapy, radiotherapy, ovarian reserve, oocyte cryopreservation, ovarian tissue cryopreservation, premature ovarian insufficiency, infertility

## Abstract

Chemotherapy and radiotherapy, among other gonadotoxic treatments, can significantly affect ovarian reserve and function, potentially leading to premature ovarian insufficiency (POI) and sterility. With the increasing survival rates among young female cancer patients, fertility preservation (FP) has become an essential aspect of cancer care. The decision to pursue FP depends on various factors, including patient age, ovarian reserve, the type of treatment, and its gonadotoxic potential. Several FP strategies are available, including oocyte, embryo, and ovarian tissue cryopreservation. While oocyte and embryo cryopreservation are the gold standard techniques, ovarian tissue cryopreservation and in vitro maturation (IVM) present viable alternatives for patients who cannot undergo ovarian stimulation or for whom stimulation is contraindicated. Despite significant advances within the FP practice, challenges remain in ensuring timely FP counseling, equitable access to services, and optimizing long-term reproductive outcomes. Continued research is needed to refine existing FP techniques, explore innovative approaches, and address ethical considerations in FP decision-making. This review explores current FP options, their clinical applications, and future directions to improve reproductive outcomes in young women undergoing gonadotoxic treatments.

## 1. Introduction

According to the World Health Organization (WHO), cancer is a leading cause of death worldwide, with 19.3 million new cases and 10 million deaths in 2020 [1,2]. In France, it was the leading cause of death in 2018, with over 150,000 fatalities [3]. Cancer remains a major public health challenge, requiring ongoing efforts in prevention, treatment, and care.

However, in recent years, the survival of cancer patients has increased significantly given the diagnostic and therapeutic advances [4]. Cancer treatments such as chemotherapy and radiotherapy have significant side-effects, particularly on ovarian function, which can lead to premature menopause and impaired fertility. Gonadotoxicity varies according to the treatment. As a result, fertility preservation (FP) has become a major component in the management of cancer patients [5].

Preserving fertility in cancer patients is essential for their quality of life, as reduced fertility can significantly affect their mental and emotional well-being [4]. Fertility preservation offers the chance to conceive later, helping alleviate distress.

Considering the potential gonadotoxic effects of cancer treatments in young women, establishing clear criteria for FP is critical. This review examines the effects of cancer treatments on ovarian function and explores the available FP strategies for young women. It focuses on the most common cancers affecting women of reproductive age, including breast cancer, hematologic malignancies, gynecological cancers, and thyroid cancer. The review discusses the gonadotoxicity of treatments such as chemotherapy (including alkylating agents and platinum-based compounds), radiotherapy, and targeted therapies and their impact on ovarian reserve. Additionally, it evaluates FP strategies recommended for each condition, including oocyte and embryo cryopreservation, ovarian tissue cryopreservation, and the use of gonadotropin-releasing hormone (GnRH) analogs, emphasizing their indications, feasibility, and efficacy in various oncological contexts.

## 2. Cancer Treatments

Cancer treatment is based on a multidisciplinary approach, combining several therapeutic modalities. The main treatments include chemotherapy, radiotherapy, and surgery, with the more recent addition of targeted, hormonal, and immune therapies.

### 2.1. Chemotherapy

The gonadotoxicity of chemotherapy depends on the class of chemotherapy and the dosage used but also on the patient’s age and fertility baseline status, most importantly ovarian reserve (OR), before starting treatment [6]. The OR represents the stock of follicles present in a woman’s ovaries at a given age. It is a quantitative marker that predicts the response to ovarian stimulation with gonadotropins. The OR is assessed by measuring the anti-mullerian hormone (AMH) and with a pelvic ultrasound to count the number of antral follicles (AFC) in the ovaries [7].

In the short term, chemotherapy affects growing follicles (pre-antral and antral follicles) causing a drop in AMH and chemo-induced amenorrhea, even with chemotherapies of low toxicity [6]. Once chemotherapy is over, AMH levels increase as follicles resume their growth, stabilizing approximately 12 months post-treatment [8]. Thus, a reassessment of OR should be conducted after 12 months post-treatment.

However, the destruction of reserve follicles will impact OR over the longer term. One theory that may explain the impact of chemotherapy on reserve follicles is the “burn-out effect” [6]. Physiologically, the recruitment of primordial follicles is regulated by highly controlled mechanisms that ensure a sustainable balance between growing (notably via PI3K/Akt/mTOR pathway) and quiescent follicular pools (notably via AMH secretion by growing follicles playing its role of negative feedback in the activation of follicular growth). Chemotherapy is thought to prevent the secretion of AMH and activate the PI3K/Akt/mTOR pathway. Akt activation is regulated by the balance between PTEN and PI3K, which results in varying degrees of Akt phosphorylation and consequently the activation of a number of pathways (such as FOXO and mTOR). Stimulation of the Akt/FOXO and mTOR pathways accelerates primordial follicle recruitment and growth [9,10,11]. The result is an increase in the number of growing follicles, which will enter apoptosis under the effect of chemotherapy and thus lead to the depletion of the follicular pool [12]. If all the reserve follicles are affected, this leads to premature ovarian failure (POI). If the destruction of the reserve follicles is not complete, early menopause may occur a few years later [6].

Chemotherapy impacts ovarian function via other mechanisms: inflammation created in the ovarian stroma leads to cortical fibrosis and interstitial cell apoptosis, while oxidative stress induced by treatments causes the thickening and hyalinization of ovarian blood vessels, leading to the narrowing or even obliteration of ovarian vessels [6].

Chemotherapies can be classified according to their mode of action.

#### 2.1.1. Direct Action on DNA

Alkylating agents are the oldest class of anticancer agents; the first alkylating agent was marketed by the FDA (Food and Drug Administration) in 1949 [13]. They form covalent bonds (alkylation) with DNA, inhibiting DNA replication and transcription. This class of chemotherapy is the most gonadotoxic [6,14,15]. There are several alkylating agents, such as cyclophosphamide, ifosfamide, busulfan, and procarbazine, each of which has a greater or lesser impact on ovarian reserve (OR) and fertility. In order to standardize the cumulative doses of the various alkylating agents, a formula has been developed to calculate the cyclophosphamide equivalent dose (CED) in mg/m^2^ [16]. The formula for calculating the CED is as follows:

1 × (cumulative dose of cyclophosphamide in mg/m^2^) + 0.244 × (cumulative ifosfamide dose in mg/m^2^) + 0.857 × (cumulative dose of procarbazine in mg/m^2^) + 8.823 × (cumulative busulfan dose in mg/m^2^) + 14.286 × (cumulative chlorambucil dose in mg/m^2^) + 15 × (cumulative dose of carmustine in mg/m^2^) + 16 × (cumulative dose of lomustine in mg/m^2^) + 40 × (cumulative melphalan dose in mg/m^2^) + 50 × (cumulative thiotepa dose in mg/m^2^) + 100 × (cumulative dose of chlormethine in mg/m^2^).

This formula is used to assess the gonadotoxicity of chemotherapies. It is a crucial criterion to assess the need for FP, as the CED dose entails a risk of premature ovarian insufficiency (POI) which varies with age. The average doses causing amenorrhea at 40, 30, and 20 years of age are 5.2 g, 9.3 g, and 20.4 g, respectively [17]. Other authors have found a significant risk of infertility only in patients receiving doses of alkylating agents higher than 7121 mg/m^2^ [18]. However, the teams involved in fertility preservation must keep in mind that CED as a comparative measure presents limitations. It offers a comparable index for myelotoxicity but is less consistent for other long-term effects such as infertility [19].

Platinum salts act like alkylating agents by binding to DNA and causing breaks during replication. The fertility risk of this class of chemotherapy is intermediate [6]. Cisplatin and carboplatin are platinum salts with very broad indications.

Splitting agents like bleomycin inhibit DNA synthesis and transcription by causing DNA breaks. This class of chemotherapy is generally considered to carry a low risk of infertility [6].

Topoisomerase inhibitors manage DNA supercoiling. Irinotecan and topotecan, inhibitors of topoisomerase I, have a low risk of infertility. Etoposide and anthracyclines (adriamycin, epirubicin) inhibit topoisomerase II. Etoposide has a low risk of infertility, while adriamycin carries an intermediate risk [6].

#### 2.1.2. Indirect Action on DNA

Spindle poisons act on the mitotic spindle tubulin. Taxanes, including docetaxel and paclitaxel, inhibit depolymerization, whereas vinca-alkaloids, such as vincristine and vinblastine, inhibit microtubule polymerization with alpha-tubulin, blocking cells in metaphase and inhibiting mitosis. These molecules present an intermediate risk to fertility [6,14].

Antimetabolites inhibit metabolic processes by competing with similar endogenous compounds, disrupting nucleic acid synthesis. This class includes anti-pyrimidines (5-fluorouracil), antifolates (methotrexate), antipurics (mercaptopurine), and hydroxyurea. They present a low risk to fertility [6].

The gonadotoxicity of the various molecules is summarized in Table 1.

In 2020, ESHRE published a more detailed classification of the risk of the treatment according to the age of patients and the protocols (Table 2).

### 2.2. Other Classes of Anticancer Agents

Targeted therapies can affect growth factors, their receptors, or intracellular components. One example is monoclonal antibodies (identified by the suffix -mab). The impact of these therapies on fertility remains unknown [14].

Immunotherapy targets the patient’s immune system, enabling activated immune cells to attack cancer cells. The potential risk of infertility associated with this kind of treatment remains unknown [14].

Hormone therapy is used when tumor cells express hormone receptors, preventing hormonal stimulation of the tumor. These treatments are not gonadotoxic, but because of their fetotoxicity, pregnancy is contraindicated during the entire course of treatment, which will postpone the pregnancy project for several years and lead to a physiological decline in ovarian reserve during the course of treatment [21,22]. That is why we can offer FP with those treatments.

### 2.3. Radiotherapy

The effect of radiotherapy on fertility is influenced by factors such as the patient’s age, the radiation dose delivered to reproductive organs (ovaries, uterus), the site of irradiation (pelvic, abdominal, hypothalamic–pituitary), and other associated treatments. It affects ovarian reserve, as well as the uterus and ovarian endocrine function depending on the location of treatment [23].

The threshold for ovarian radiosensitivity is low: a dose of 2 Gy is sufficient to reduce the reserve follicle count by half [23]. The decline in the number of primordial follicles is proportional to the size of the follicle pool. Consequently, for a given radiation dose, the younger the patient is at the time of radiotherapy, the later the onset of premature menopause will be. The doses that result in premature ovarian insufficiency in 97.5% of cases immediately after treatment are 20.3 Gy at birth, 18.4 Gy at 10 years, 16.5 Gy at 20 years, and 14.3 Gy at 30 years [23]. However, as soon as 6 Gy is administered to the ovaries, patients are considered to be at high risk of infertility [14,23].

Radiotherapy can have deleterious effects on uterine function. Myometrial fibrosis, altered vascularization, and reduced volume can occur with as little as 14 Gy exposure. This increases obstetrical risks, including spontaneous miscarriage, intra-uterine growth retardation, and premature deliveries [23,24].

Hypothalamic–pituitary irradiation may lead to a central hormonal deficit, which depends on the dose received and the time elapsed since irradiation, resulting in a risk of secondary POI [25,26]. Hypothalamic–pituitary irradiation may be required in various cases. For instance, in the case of brain tumors, treatment involves hypothalamic–pituitary irradiation with doses of up to 60 Gy. In the case of conditioning prior to bone marrow transplantation for relapsed leukemia or high-risk leukemia in children, whole-body irradiation will also affect the hypothalamic–pituitary zone, with doses ranging from 10 to 14 Gy [23].

## 3. Fertility Preservation Techniques

**Figure 1:**
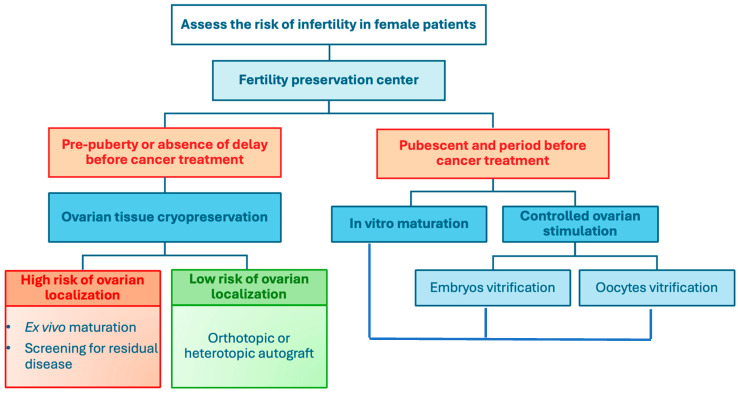
FP techniques in the oncological setting for women (adapted from [27]).

Various FP techniques are available for women (Figure 1). The choice depends on several criteria: the patient’s age, OR before treatment, the indication for FP, presumed gonadotoxicity, previous and upcoming treatments, and the time available before starting treatments [14]. Other factors, such as the patient’s preference to have children and to perform FP, the risk of cancer relapse requiring more toxic treatments, and the potential delay in parental plans due to anti-cancer treatments (leading to a physiological decrease in ovarian reserve), may also influence the decision.

Female FP options include oocyte vitrification after ovarian stimulation or after in vitro maturation, embryo cryopreservation, and ovarian tissue cryopreservation with the possibility of ex vivo oocyte maturation (Figure 1).

### 3.1. Oocyte Vitrification After Ovarian Stimulation

Ovarian stimulation will last 12 to 15 days with daily injections of exogenous FSH. Ultrasound and the biological monitoring of follicular maturation allow the FSH doses to be adjusted to the ovarian response of each patient. When the follicles are mature, ovulation is triggered using a GnRH (gonadotropin-releasing hormone) agonist or recombinant hCG (human chorionic gonadotropin). Ultrasound-guided transvaginal ovarian puncture is performed 36 h after the beginning of ovulation, under local or general anesthesia [28]. Cumulus-oocyte complexes are decoronized (removal of granulosa cells) to retain only the mature oocytes. Oocytes are then immersed in liquid nitrogen at −196 °C [29,30].

In an oncological context, the live birth rate for patients under 35 years with 5 vitrified oocytes is 9.1%, while with 10 vitrified oocytes the live birth rate is 42.9%. In patients over the age of 35 years, the live birth rate is 11.1% with 4 vitrified oocytes and 43.4% with 10 vitrified oocytes [31]. The average live birth rate following egg vitrification in the context of oncological FP is 32% according to the meta-analysis by Fraison et al. [32]. However, oocyte vitrification has an 80–90% survival rate after thawing and requires fertilization post-thawing, adding uncertainty to success rates.

### 3.2. Embryo Vitrification

Embryo vitrification was commonly used when oocyte vitrification was not allowed in France and egg slow-freezing had poor results. According to Fraison et al. [32], the live birth rate after embryo vitrification for oncological FP is 41%, slightly higher than that for oocyte vitrification. This method shows similar outcomes as those for infertile couples in medically assisted procreation (MAP), with better survival rates post-thawing (95%) and higher pregnancy chances than oocyte vitrification [33]. However, since the 2011 bioethics law in France permitting egg vitrification, embryo vitrification is no longer the first choice for FP. Indeed, unlike oocyte vitrification, embryo freezing preserves the couple’s fertility rather than the woman’s alone. In some countries, such as France, strict legal frameworks prevent the use of embryos if the spouse passes away or the couple separates, effectively eliminating all the woman’s FP options [33]. In contrast, other jurisdictions, such as Belgium, allow posthumous reproduction under specific conditions, provided that prior consent was given and within a legally defined timeframe. These legal and ethical differences underscore the significance of considering not only the medical but also the regulatory aspects of embryo vitrification when advising patients on FP strategies.

### 3.3. In Vitro Maturation

In vitro maturation (IVM) is a technique that can be offered in case of urgent FP indication (without delay for ovarian stimulation) or in the case of contraindication to ovarian stimulation (for certain histological subtypes of breast cancer, high thrombotic risk, etc.) or when the benefit–risk balance is unfavorable (polycystic ovary syndrome and a high risk of ovarian hyperstimulation syndrome). This technique involves the collection of oocytes from small growing antral follicles, without any ovarian stimulation. It must necessarily be carried out before the start of chemotherapy [34]. In the laboratory, the oocytes collected are placed in an enriched culture medium with IVM medium and patient serum or human serum albumin (HSA) with FSH and LH at 37 °C for approximately 36 h to mature them. Oocyte maturation is evaluated between 24 and 48 h later. All mature oocytes are vitrified [35,36].

Thresholds of 20 antral follicles and 3.7 ng/mL AMH were established for achieving the cryopreservation of at least 10 mature oocytes following IVM [37]. Some authors state that in vitro maturation results in vitrifying half as many mature oocytes compared to oocyte vitrification following controlled ovarian stimulation [38]. According to Mayeur et al., only 10 live births have been reported following the use of oocytes or embryos obtained after IVM for cancer-related FP [39].

### 3.4. Ovarian Tissue Cryopreservation

Ovarian tissue cryopreservation (OTC) consists of a total or partial unilateral oophorectomy to freeze the ovarian cortex in which the primordial and primary immature follicles are found [40]. The ovarian cortex is isolated in the laboratory and then cut into fragments that are placed in cryotubes to be frozen after exposure to a cryoprotectant. OTC can be performed as an emergency procedure at any time during the cycle. It is indicated in cases where high-risk gonadotoxic treatments are being considered for adults under the age of 36 and for whom the start of treatment cannot be postponed but also in cases where gonadotoxic treatment has already begun because no follicles can grow and respond to ovarian stimulation in the first year post-treatment [41]. It is also the only FP technique that can be offered to prepubescent children [42].

However, this technique has drawbacks. It involves surgery with a general anesthetic in an already very demanding process. And when it comes to grafting the tissue removed, there is a risk of reintroducing the disease due to the presence of tumor cells in the graft, particularly in the case of certain pathologies that are at high risk of ovarian metastasis such as leukemia, neuroblastoma, or Burkitt’s lymphoma [41]. Different techniques detect traces of the disease, depending on the type of pathology. Recent advancements include immunohistochemistry targeting specific markers, PCR-based pathogen detection, next-generation sequencing (NGS), and xenotransplantation models, which have improved screening sensitivity. However, as these techniques for detecting residual disease are still not fully reliable, the decision to re-implant ovarian tissue is made with a specialized multidisciplinary consultation meeting.

After recovery, an autograft of previously frozen ovarian tissue can restore endocrine function and fertility [40,43,44]. The transplant can be performed orthotopically or heterotopically and can be performed in one or two stages [45,46]. The rate of restoration of ovarian function varies between 85.2% and 95% according to the authors [43], with a return of menstruation within 4.5 ± 2.2 months after the transplant [42]. In addition, recent meta-analyses by Ramirez et al. and Khattak et al., have shown an increase in estradiol from 101.6 pmol/L to 522.4 pmol/L after ovarian tissue transplantation and a decrease in FSH from 66.4 IU/L before transplantation to 14.1 IU/L after transplantation. The average duration of graft function is 2.5 years, ranging from 0.7 to 5 years [44,47]. According to Fraison et al., the live birth rate for spontaneous pregnancies after autotransplantation is 33%, and the rate for live births after autotransplantation followed by IVF is 19% [32].

### 3.5. Combination of Different FP Techniques

Several ways of combining FP techniques have been described. Ovarian stimulation for oocyte vitrification followed by laparoscopy on the day of the puncture for an OCT has been reported by some authors [48]. The reverse strategy, with CTO as a first step followed by ovarian stimulation for oocyte vitrification can also be considered, with stimulation starting one or two days before the laparoscopy [49]. Performing laparoscopy prior to ovarian stimulation may be advantageous, as it avoids surgery on hyper-vascularized and enlarged ovaries, thereby reducing the risk of bleeding and surgical complications during the procedure. It is also possible to remove ovarian tissue in order to puncture immature oocytes ex vivo for IVM and then cryopreserve the remaining ovarian tissue [50,51].

### 3.6. Surgical Techniques for FP

Conservative surgery is used for gynecological cancers and is defined by the preservation of the uterus and at least one of the adnexa after full staging to confirm that the tumor is localized and has not spread. Patients who have conservative surgery must be monitored regularly and over the long term to prevent recurrence. Once the parental project is complete, radical surgery is recommended [52]. This type of surgery is proposed for localized ovarian, cervix, or endometrium tumors at an early stage.

### 3.7. GnRH Agonists

GnRH agonists have been proposed as a strategy to preserve ovarian function during chemotherapy. Their protective effect is thought to result from the suppression of gonadotropin secretion, which reduces ovarian activity. Reduced follicular recruitment may, in turn, lead to a reduction in the number of growing follicles and thus to a reduction in chemotherapy-induced ovarian damage. But GnRH agonists may have side effects due to induced hypoestrogenism (hot flushes, asthenia, libido problems, vaginal dryness, etc.). Additionally, GnRH agonists may have a direct protective effect on ovarian follicles through mechanisms that are still under investigation. The use of GnRH agonists remains highly controversial, as existing literature does not provide conclusive evidence that these agonists offer beneficial effects on ovarian reserve or fertility [53]. GnRH agonists may be prescribed for the purpose of inducing therapeutic amenorrhea in the case of thrombopenic chemotherapy and/or for long-term parenteral contraceptive effects. The National Cancer Institute’s (InCA) latest recommendations affirm that “GnRH agonists cannot be recommended as a method of fertility preservation” [53].

## 4. Cancers and Fertility Preservation in Young Women

The incidence of cancer in women aged between 15 and 39 worldwide is 56.2 per 100,000 [54]. This rate is two times higher than for men of the same age. The three most common cancers are breast, thyroid, and cervical, followed by lymphoma, ovarian cancer, and leukemia [4,54].

### 4.1. Breast Cancer

Breast cancer is the world’s leading cancer in terms of incidence and mortality, with 2.3 million new cases and 685,000 deaths [55]. In France, between 1990 and 2023, the annual number of new cases of breast cancer had doubled from 30,000 to 61,000 by 2023 [56], but mortality fell by 1.6% a year between 2010 and 2018 [57]. The median age at diagnosis is 64. However, breast cancer is the most common cancer in women of childbearing age, with 7% of breast cancers diagnosed in patients under 40 [58]. With advances in medical techniques for cancer detection and treatment, the prognosis is now more favorable, as cancers are diagnosed and treated earlier. The 5-year relative survival rate has reached 99% for localized breast cancer [21].

The main risk factors for breast cancer are age, a personal history of breast pathology, a family history of breast cancer including genetic predisposition (mainly *BRCA1* or *2*), and a history of high-dose thoracic radiotherapy (as part of treatment for Hodgkin’s lymphoma, for example) [59].

#### 4.1.1. Treatments

Breast cancer treatment will depend on the stage of the cancer, the hormone receptor, and HER2 status [55].

Breast-conserving surgery is suitable for most patients with breast cancer. Often followed by radiotherapy, this technique is usually accompanied by reconstruction surgery [55]. In cases where primary breast conservation is not possible, nipple-preserving mastectomy and skin-preserving mastectomy offer good results both oncologically and aesthetically [55].

After breast-conserving surgery, radiotherapy significantly reduces the risk of breast cancer recurrence and mortality [55]. Depending on the prognosis and aggressiveness of the tumor, radiotherapy may be complete or partial, with equivalent results [55]. Generally, the doses used for local and/or regional adjuvant irradiation are 45 to 50 Gy in 25 to 28 fractions with a boost dose of 10 to 16 Gy [55].

For hormone-receptor-positive, HER2-negative breast cancers, several approaches are possible. The luminal A subtype is more often low-grade and has a good prognosis; in this case, chemotherapy is not systematic and is discussed according to the response to hormone therapy. The luminal B subtype is more often high-grade, with a poorer response to hormone therapy, and therefore requires chemotherapy. Neoadjuvant chemotherapy can be used to reduce tumor mass prior to surgery to facilitate surgical intervention. Adjuvant chemotherapy is most often required to reduce the risk of recurrence after surgery [55].

For HER2-positive breast cancer, it is the addition of targeted therapy to chemotherapy that has revolutionized prognosis, notably trastuzumab. This has reduced mortality by around a third. Neoadjuvant chemotherapy is often indicated for patients with stage II-III cancer to reduce tumor size before surgery. Adjuvant chemotherapy is systematically combined with HER2-targeted therapy [55].

For patients with triple-negative breast cancer, treatment is mainly based on the use of neoadjuvant chemotherapy. This reduces tumor size prior to surgery and allows for the evaluation of therapeutic response (a prognostic factor) [55].

The chemotherapies used in breast cancer protocols are mainly based on anthracyclines and taxanes combined with cyclophosphamide (most often EC protocol, epirubicin, and cyclophosphamide) [55].

Hormone therapy is systematically administered to hormone-receptor-positive patients for 5 to 10 years after chemotherapy, depending on the aggressiveness of the tumor. Tamoxifen is prescribed alone or in combination with an aromatase inhibitor (exemestane, letrozole, or anastrazole), depending on the type of tumor [60]. These treatments do not pose a risk to ovarian function, but as they are toxic to the fetus, their administration contraindicates pregnancy and therefore postpones the pregnancy project [21,22]. There is therefore a physiological decline in OR with advancing age. In specific cases, especially for patients with a high risk of recurrence, a GnRH analog may be administered in combination with other treatments. This induces a temporary suppression of ovarian function, but may also lead to a slight bias in AMH measurement (mean decrease of 0.26 ng/mL found by Yin et al. after 3 months of GnRH analog treatment) [22].

Targeted therapies like HER2, PARP, CDK4/6, and PD-1/PD-L1 inhibitors are used to treat breast cancer. However, their impact on fertility is not well-studied [55].

These different treatments have varying degrees of impact on fertility and require appropriate fertility preservation management.

#### 4.1.2. Fertility Preservation

Oocyte cryopreservation after ovarian stimulation is the most widely used FP technique for managing patients with breast cancer [61]. To be able to propose it, several criteria must be taken into consideration: there must be sufficient time to carry out ovarian stimulation, and the patient’s clinical condition must allow it. But ovarian stimulation also requires the agreement of the oncology team because of the secondary induced hyperestrogenism and its potential risks, particularly for hormone-receptor tumors. The addition of aromatase inhibitors (letrozole) to standard stimulation protocols can reduce peak estradiol levels [62,63]. Letrozole is used in many countries but has no marketing authorization in France for this indication. Tamoxifen (a selective estrogen receptor modulator) can be used instead of letrozole to reduce peak estradiol levels [64]. For the same reasons, GnRH agonists are used for ovulation induction rather than hCG [61,65].

Therapeutic urgency does not always allow ovarian stimulation to be started in the follicular phase. The “random start” protocol enables stimulation to be started at any time during the ovarian cycle. Compared with conventional stimulation, random start tends to require higher doses of gonadotropins and a longer duration, but no significant difference has been observed in the quantity of oocytes punctured or vitrified compared with conventional techniques [61].

In order to obtain a greater number of oocytes over a shorter period of time, it is possible to combine two stimulations and two oocyte punctures in the same menstrual cycle, during the follicular and luteal phases [66]. In this way, the first oocyte puncture is performed after the first stimulation phase, and the second stimulation begins the day after the first puncture. The number of punctured oocytes, mature oocytes, and embryos resulting from the double stimulation method is significantly higher than in a conventional cycle with a single stimulation [66].

In vitro maturation (IVM) is used in cases of therapeutic emergencies and insufficient time for ovarian stimulation. Given the low yield of this technique, a good OR is required to obtain a reasonable number of oocytes [37].

Ovarian tissue cryopreservation (OTC) is mainly used in patients with a therapeutic emergency, leaving no time for ovarian stimulation [67,68]. In the context of breast cancer, when ovarian stimulation is not possible, OTC has rarely been proposed until now but is more and more considered due to the low yield of mature vitrified oocytes from IVM. OTC also has the advantage of being able to restore endocrine function after frozen tissue transplantation and offers the possibility of achieving spontaneous pregnancies [67,68].

#### 4.1.3. BRCA-Mutated Patients

Patients with a *BRCA1* or *BRCA2* gene mutation are at greater risk of developing breast cancer, often at an early age. They are also at greater risk of developing contralateral cancer and ovarian cancer. The cumulative risk of developing breast cancer at age 70 is 51–75% with a median age of onset of 40 years for patients with the *BRCA1* mutation, and 33–55% with a median age of onset of 43 years for patients with the *BRCA2* mutation. This implies extensive surveillance, prevention by prophylactic surgery, and therefore FP strategies specific to this patient population [69]. To reduce the risk of ovarian cancer, prophylactic bilateral adnexectomy is strongly recommended for women with *BRCA1* mutations before the age of 40 and can be deferred until the age of 45 for women with *BRCA2* mutations [69]. This procedure considerably reduces the risk of ovarian cancer but leads to early menopause.

Furthermore, these patients will potentially receive high-risk gonadotoxic chemotherapy if the cancer occurs, and they may also receive hormone therapy. All of this will have the effect of postponing their pregnancy plans and thus increasing the risk of POI. For these reasons, FP should be considered for all *BRCA-mutated* patients, even if they have not yet developed cancer. Oocyte vitrification is the FP technique most often proposed in this context and several cycles of ovarian stimulation can be performed since there is no rush to start treatment [70]. OTC provides an alternative option for FP, particularly for younger patients who cannot undergo ovarian stimulation. However, the potential risk of malignant cell reintroduction upon ovarian tissue transplantation is a significant concern in *BRCA1/2* patients who may develop occult ovarian cancer. Additionally, the lower follicular density observed in *BRCA* carriers may affect the efficacy of OTC compared to non-carriers. Careful patient selection, thorough histopathological and molecular screening of ovarian tissue, and long-term follow-up are required to assess the safety and feasibility of this approach [71]. Furthermore, *BRCA*-mutated patients are advised to undergo prophylactic oophorectomy around the age of 40 to reduce their risk of ovarian cancer. If ovarian tissue transplantation (OTT) is performed previously, the need for subsequent oophorectomy could pose surgical challenges, making the procedure more complex and potentially compromising its feasibility.

Further, genetic counseling is indicated since patients must be informed of the risk of transmitting the gene to their offspring.

Furthermore, *BRCA* mutations are associated with reduced OR. Wang et al. found a statistically significantly lower AMH level in *BRCA1* mutated patients [72], and Lin et al. showed that the age of menopause in *BRCA1/2* mutated patients was earlier [73]. Also, Oktay et al. observed that *BRCA1*-mutated patients have fewer oocytes at oocyte puncture after ovarian stimulation compared with non*-BRCA1-*mutated patients [74]. Finally, Grynberg et al. demonstrated that women with *BRCA* mutations without cancer have a significantly lower primordial follicle density than non-mutated women, as well as a higher rate of DNA double-strand breaks in primordial follicle oocytes [70]. These findings suggest that *BRCA*-mutated patients may have a naturally compromised OR even before exposure to gonadotoxic treatments, reinforcing the importance of early FP strategies in this population.

### 4.2. Thyroid

Thyroid cancer affects women three times more than men and can occur at any age. The median age at diagnosis is 50, and it is the third most common cancer in women between the ages of 25 and 45 [75]. The main risk factors are exposure to ionizing radiation (particularly during childhood) and iodine deficiency [75]. Late menarche or early menopause are also risk factors for papillary thyroid carcinoma. On the other hand, the use of oral contraceptives, hormone replacement therapy, and breastfeeding have been found to be protective factors [76,77].

#### 4.2.1. Treatment

Surgery is the first-line treatment for thyroid cancer [75]. Thyroidectomy can be either total or partial (lobectomy), depending on tumor size, lymph node involvement, or presence of metastasis. Iratherapy, or internal radiotherapy with iodine-131, is indicated according to the risk of recurrence for well-differentiated tumors. In rare cases, external radiotherapy may be indicated [77].

#### 4.2.2. Fertility Preservation

Thyroid cancer treatments do not pose a risk to fertility, so FP is not recommended [14,76,78].

### 4.3. Cervical Cancer

Cervical cancer is one of the most common cancers affecting women worldwide and is particularly prevalent in sub-Saharan Africa, Latin America, the West Indies, and Southeast Asia [79]. Over the last few decades, the incidence and mortality of this cancer have fallen considerably in industrialized countries, thanks to the introduction of cervico-uterine smear screening and a vaccine [79]. In France, there are 2850 new cases per year, and 46% of patients are under 40 at diagnosis [80]. Persistent infection with a high-risk HPV (human papillomavirus) is the main risk factor for cervical cancer. There are several types, but the most common are HPV-16 and HPV-18, which are responsible for over 80% of cervical cancers [80]. Other risk factors have been identified, such as early sexual debut, multiple partners, smoking, multiparity, the use of oral contraception, and low socio-economic status [79].

#### 4.3.1. Treatment

Treatment will depend on the stage of the cancer. The standard treatment is hysterectomy with lymphadenectomy [81]. However, conization may be proposed for early stages IA1 and IA2 and trachelectomy for stages IB1 without lymph node involvement (involves removing the cervix while preserving the uterus). For more advanced stages, hysterectomy with lymphadenectomy should be accompanied by brachytherapy and chemotherapy (cisplatin) as appropriate [82].

#### 4.3.2. Fertility Preservation

FP can only be proposed for early stages with tumors less than 2 cm in size and localized, without lymph node involvement. In these cases, conservative surgery can be performed as described above, by conization or trachelectomy depending on the stage, enabling spontaneous fertility to be preserved. In all other cases, hysterectomy is indicated and no FP technique can be proposed [82].

### 4.4. Hodgkin’s Lymphoma

Hodgkin’s lymphoma has an incidence of 3.2 per 100,000 inhabitants per year in France. There are two peaks in the onset of the disease: in young adults under the age of 30, and then in older individuals, around the age of 70. The median age at diagnosis is 27 [83,84].

#### 4.4.1. Treatment

There are several polychemotherapy protocols depending on the stage of the lymphoma and the therapeutic response [85,86]: the ABVD protocol (doxorubicin, bleomycin, vinblastine, dacarbazine), the BEACOPP protocol (bleomycin, vincristine, etoposide, cyclophosphamide, doxorubicin, procarbazine, prednisolone), and the BEACOPDac protocol (bleomycin, vincristine, etoposide, cyclophosphamide, doxorubicin, dacarbazine, prednisolone). Dacarbazine has replaced procarbazine, with equivalent results but less impact on fertility [86,87]. If the first line of therapeutics fails to control the disease, or in the event of relapses, there is an indication for hematopoietic stem cell autotransplantation after conditioning, which requires extremely gonadotoxic protocols such as BEAM (carmustine, etoposide, cytosine arabinoside, melphalan) [84].

#### 4.4.2. Fertility Preservation

The fertility preservation strategy depends not only on the chosen chemotherapy protocol but also on the patient’s age, ovarian reserve, clinical condition, personal motivation, and the urgency of starting anti-cancer treatments. If the chosen protocol is ABVD or BEACOPDac, as these treatments are not highly gonadotoxic, there is no indication for FP [88,89]. If it is BEACOPP, FP is indicated due to the significant gonadotoxicity of the protocol [88,90,91]. Oocyte vitrification after ovarian stimulation or OTC may be discussed, depending on the time available before the start of treatment, the patient’s clinical condition, and her personal motivation. If an allograft is needed, OTC should be proposed in view of the significant gonadotoxicity of conditioning treatments and the therapeutic urgency [42,88]. If the patient has already received chemotherapy in the past year, OTC is the only available option.

### 4.5. Non-Hodgkin’s Lymphoma

Non-Hodgkin’s lymphomas (NHLs) represent a heterogeneous group of malignant neoplasia. Their incidence has increased in recent decades, especially in industrialized countries. In 2020, around 545,000 new cases of NHL were diagnosed worldwide [92]. The frequency of different lymphoma subtypes varies according to geographical area. For example, T lymphoma is much more common in East Asia (associated with the HTLV virus) [93]. In France, the incidence of NHL was 27,000 new cases per year in 2018, with a median age of 60 [94]. The risk factors for NHL remain unclear. However, it seems that infection with HCV (hepatitis C virus) or EBV (Epstein–Barr virus) or being immunosuppressed are risk factors [95].

#### 4.5.1. Treatment

The treatment used for most NHL subtypes is R-CHOP, a combination of CHOP polychemotherapy (cyclophosphamide, doxorubicin, vincristine, and prednisone) and anti-CD20 monoclonal antibodies (rituximab). Depending on the subtype of NHL, Brentuximab–Vedotin, tyrosine kinase or BCL-2 inhibitors, immunotherapy, radiotherapy, or hematopoietic stem cell autotransplantation (refractory or relapsed NHL) may be used [93,96].

#### 4.5.2. Fertility Preservation

FP management depends on the treatments envisaged, the delay before the start of treatment, the patient’s age, her OR, and her clinical condition. If R-CHOP cures are envisaged, oocyte vitrification after ovarian stimulation may be proposed because of the intermediate risk of infertility [14,96]. In the case of autografts, conditioning protocols are highly gonadotoxic [14,96] and an OTC may be proposed.

Concerning other treatments, as there are no data on gonadotoxicity, no FP management is recommended [96].

### 4.6. Ovarian Cancer

Ovarian tumors are all proliferative processes, benign or malignant and cystic, solid, or vegetative in appearance, whose growth is not directly linked to hormonal dysfunction. There are three types of ovarian tumors: benign, malignant, and borderline [97,98].

#### 4.6.1. Malignant Tumors

Malignant tumors are rare tumors, accounting for 3% of all female cancers. The second most common gynecological cancer after endometrial cancer, it has a poor prognosis, with a 5-year survival rate of just 43% in France [99]. This is partly explained by the fact that in 75% of cases, the diagnosis is made at an advanced stage. The median age at diagnosis is 70, with only 3% to 17% of patients under the age of 40. In these younger patients, the 5-year survival rate is 94–98% [100,101,102]. Established risk factors for ovarian tumors include nulliparity, early menarche, and late menopause. Pregnancy, breastfeeding, and estrogen–progestogen contraception are considered to be protective factors [97].

##### Treatments

Treatment varies according to FIGO stage. For stages IA or IB, surgery alone is the rule. It includes infra-colic omentectomy, pelvic and lombo-aortic lymph node dissection, peritoneal biopsies, the removal of any peritoneal fluid or ascites, and, finally, total hysterectomy and bilateral salpingo-oophorectomy. The prognosis for these localized stages is excellent, with over 90% survival at 5 years and rare contralateral relapse (0 to 2.5% of cases) [97,102,103]. For more advanced stages IC, II, III, and IV, surgery is the same but chemotherapy with carboplatin and paclitaxel must be added [97,102].

##### Fertility Preservation

FP in malignant ovarian cancer concerns very few patients. In fact, only 9.8% of women with malignant ovarian cancer are under 45, and 1.2% are eligible for conservative surgical treatment [104].

For low-grade stage IA serous, mucinous, or endometrioid tumors, conservative surgical treatment may be proposed after complete staging, including curettage for endometrioid and mucinous subtypes. This involves unilateral adnexectomy with conservation of the uterus and contralateral adnexa [102,105,106,107]. Patients should be made aware of the 6–13% risk of recurrence in the contralateral ovary, which implies regular follow-up of patients treated conservatively [105]. The prognosis remains excellent, with a 5-year survival rate of over 90%. Patients should also be aware that unilateral adnexectomy leads to a reduction in ovarian reserve and therefore a risk of premature ovarian failure [103].

For low-grade stage IA epithelial clear cell carcinomas, conservative surgery is discussed on a case-by-case basis. For high-grade IA, IC1, or low-grade IC2, bilateral adnexectomy with conservation of the uterus may be proposed [102].

Conservative surgery of the uterus is not recommended for epithelial cancer extending beyond the ovaries, whatever the histological type.

To date, there is no recommendation for ovarian tissue cryopreservation or ovarian stimulation on the contralateral ovary or IVM or GnRH analog [100,102,107]. Any history of serous, endometrioid, mucinous, or clear-cell tumors definitely contraindicates ovarian stimulation, given the lack of data in the literature [100,102]. Ovarian stimulation can only be discussed in the case of low-grade mucinous tumors after discussion at the Multidisciplinary Team Meeting.

#### 4.6.2. Ovarian Germ Cell Tumors

Ovarian germ cell tumors are rare malignant tumors (5% of all ovarian malignancies). They present some notable differences from epithelial tumors. Mature teratomas (95% of germ cell tumors) are the most common unilateral benign tumors in young girls. Other histological types exist but are very rare [108]. These tumors are almost always unilateral at the time of diagnosis and usually manifest themselves in adolescence as abdominal pain, a pelvic mass, fever, or metrorrhagia. The diagnosis is therefore made earlier, and the prognosis is excellent. In all cases, patients of childbearing age may be offered conservative surgery with unilateral oophorectomy, followed by adjuvant chemotherapy according to the BEP protocol (bleomycin, etoposide, and cisplatin) [109]. FP by ovarian stimulation before treatment is not recommended, but it is recommended to perform FP after tumor treatment due to the high risk of contralateral relapse [109].

#### 4.6.3. Malignant Tumors of the Sex Cords and Stroma of the Ovary

These malignant tumors of the ovary are also rare ovarian tumors. They arise from granulosa, theca, and Sertoli and Leydig cells [110]. They produce sex steroids. They can occur at any age but most often occur in young women. Given that 70% of patients are diagnosed at a localized stage, the prognosis is excellent, with a 5-year survival rate of 95% [109]. There are different histological forms: malignant Sertoli–Leydig tumors, malignant granulosa tumors, malignant steroid cell tumors, and gynandroblastoma. Clinically, abdominal pain and gastrointestinal symptoms are also present. However, the production of virilizing sex hormones in 50% of patients is characteristic, leading to precocious puberty, virilization, or primary or secondary amenorrhea [110]. The standard treatment remains surgery with total hysterectomy and bilateral salpingo-oophorectomy. Conservative surgery by unilateral oophorectomy may be proposed to patients with localized stage I disease who wish to preserve their fertility. Ovarian stimulation may be discussed on a case-by-case basis and only in patients with infertility [109].

### 4.7. Acute Leukemia

Acute leukemias (ALs) are hematological malignancies characterized by the clonal expansion of bone marrow cells blocked at an early stage [111]. They are rare pathologies, with around 2000 new cases per year in France [112]. There are two types of AL. Acute myeloid leukemia (AML), more common in the elderly, with a median age at diagnosis of 65, and acute lymphoblastic leukemia (ALL), more common in children (accounting for a third of childhood cancers) but found at all ages [111].

#### 4.7.1. Treatment

The aim of leukemia treatment is to achieve remission and prevent relapse of the disease. The main treatment is chemotherapy. For AML, it is based on anthracyclines and cytosine–arabinoside. For ALL, vincristine, asparaginase, and methotrexate are used [111]. There are new targeted therapies currently being developed, as well as immune therapies (CAR T cells) that look promising [111,113,114]. Hematopoietic stem cell transplantation after conditioning is usually reserved for relapsed patients (total body irradiation or chemotherapy containing alkylating agents) [111,113,114].

#### 4.7.2. Fertility Preservation

In the first-line treatment of AL, due to the low risk these molecules pose to fertility, FP is not required [14,115]. Nevertheless, when hematopoietic stem cell transplantation is envisaged, myeloablative conditioning is necessary, usually with a high gonadotoxic risk. An OTC may then be considered [116]. However, ALs have a high risk of ovarian tumor infiltration. Cryopreservation of ovarian cortex should be carried out after complete remission, if possible just before conditioning, to minimize residual disease. Even in the case of favorable residual disease detection tests, there is still a risk of disease relapse to consider. [116]. As a result, patients must be properly informed about the great possibility that they might never have the authorization to reuse their OTC. For this reason, fertility preservation by oocyte vitrification after ovarian stimulation at diagnosis may be discussed if the time required to start chemotherapy is compatible with ovarian stimulation.

### 4.8. Melanoma

The incidence of melanoma varies according to latitude and the pigmentary characteristics of the population. In fact, the Caucasian phenotype with fair skin is more at risk of developing melanoma. Incidence is therefore highest in Australia and lowest in Asian and African countries [117]. In France in 2023, 17,922 new cases of cutaneous melanoma were diagnosed, responsible for 1980 deaths in 2018 [118]. This represents 4% of all incident cancers and 1.2% of cancer deaths in France. The median age at diagnosis is 55. Survival will depend on the stage at the time of diagnosis, with a 5-year survival of 98% in the localized stage, 62% in the locoregional extension stage, and 15% in the metastatic stage [118,119]. The main risk factor is exposure to ultraviolet (UV) radiation, particularly a history of sunburn in childhood. Smoking and lung cancer are also risk factors [117]. A familial predisposition is found in 5 to 12% of cases [117].

#### 4.8.1. Treatment

For localized melanomas, treatment is essentially surgical, and lifelong annual dermatological surveillance is recommended [117,120]. In the case of advanced or metastatic melanoma, the only therapeutic option until recently was chemotherapy, with a poor prognosis. But targeted therapies and immunotherapy (anti-PD1 or anti-CTLA-4) have emerged in recent years, transforming the prognosis of these patients [120].

#### 4.8.2. Fertility Preservation

Patients with melanoma who do not require systemic treatment are not concerned with FP, as surgery has no impact on their fertility. The impact of systemic treatments (targeted therapies or immunotherapy) on fertility in women with metastatic melanoma is not yet known as there are only data available for animal fertility [14]. Oocyte vitrification after ovarian stimulation may be proposed if the patient wishes to have FP and if the delay before the start of treatment allows it [121].

### 4.9. Brain Tumors

In France, the incidence of primary intracranial tumors is around 6000 new cases per year. In decreasing order of incidence, these include meningiomas (40%), gliomas (30%), and pituitary adenomas (10%) [94]. Glioblastomas are the most common malignant brain tumors. Brain metastases account for over 50% of intracranial tumors [122]. Malignant brain tumors are more common in men, while benign tumors, such as meningiomas, are more common in women [122].

#### 4.9.1. Treatment

Surgical resection is not always possible, depending on the extent of the tumor. When it is possible, it provides immediate symptomatic relief, and if complete, it can be curative for low-grade tumors. For high-grade tumors, radiotherapy is also used. Chemotherapy is rarely used due to the blood–brain barrier, which limits its passage and therefore its efficacy. However, it can help sensitize the tumor to radiotherapy [123].

#### 4.9.2. Fertility Preservation

FP is rarely considered at the time of the diagnosis of malignant gliomas, given the poor prognosis of these tumors. Less than 30% of patients receive information about their fertility [124]. The reluctance of oncologists to propose FP could also be explained by the progression of gliomas observed during pregnancy. However, the use of chemotherapies with a high risk of gonadotoxicity justifies proposing oocyte vitrification after ovarian stimulation in patients of childbearing age with good OR. If there is not enough time, an OTC can also be proposed. In addition, radiotherapy to the brain has a detrimental effect on the hypothalamic–pituitary axis, resulting in a centrally induced hormonal deficit and hence secondary ovarian failure, which reinforces the need to discuss FP [124,125].

### 4.10. Primary Malignant Bone Tumors

Primary bone tumors are rare and most often affect young people. They may be benign or malignant. Osteosarcoma is the most common primary malignant bone tumor and tends to affect boys, with a peak frequency between the ages of 15 and 19 [126,127]. In 15% of cases, metastases are found at diagnosis and most often in the lung. The 5-year survival rate for localized forms is 76% and 24% for metastatic forms [127,128]. Ewing’s sarcoma is the second most common primary malignant bone tumor and tends to affect children and young adults. The 5-year survival rate for localized forms is 82% and 39% for metastatic forms [128].

#### 4.10.1. Treatment

Neoadjuvant chemotherapy reduces the size of the tumor. Surgery to remove the tumor must then be performed, followed by adjuvant chemotherapy to prevent the significant risk of relapse. Surgery is usually conservative, but when the local invasion is too extensive, amputation may be necessary (this concerns less than 10% of patients) [127,129,130]. Radiotherapy may also be considered in patients with surgically unresectable tumors, or as adjuvant therapy for tumors at high risk of relapse [129]. Chemotherapy protocols for osteosarcoma include doxorubicin, cisplatin, methotrexate, and ifosfamide. In the treatment of Ewing’s sarcoma, chemotherapies include vincristine, doxorubicin, cyclophosphamide, ifosfamide, and etoposide.

#### 4.10.2. Fertility Preservation

OTC is often the only FP option because of the therapeutic urgency of starting neoadjuvant chemotherapy. However, the risk of disease re-implantation is significant, and ovarian cortex fragments must be tested to ensure that the risk of the reintroduction of residual disease is sufficiently low [128]. If the delay before the start of chemotherapy and the patient’s OR allows it, oocyte vitrification after ovarian stimulation may be proposed due to the intermediate risk of infertility with these polychemotherapies [14,131,132,133].

### 4.11. Digestive Cancers

Digestive cancers involve the entire digestive tract (esophagus, stomach, small intestine, colon, rectum, anus), as well as related organs such as the pancreas and liver. Colorectal cancer is the most common of these cancers, accounting for 14% of cancers in women. However, these cancers are more common in the elderly and rarer in women of childbearing age. Nevertheless, it seems that the incidence of colorectal cancers in people under 50 has been rising in recent years [134].

#### 4.11.1. Treatments

Excisional surgery is the standard treatment for most digestive cancers, while platinum-based chemotherapies are used either as neoadjuvant or adjuvant therapies. FOLFOX (folinic acid, 5-FU, oxaliplatin) or FOLFIRINOX (folinic acid, 5-FU, irinotecan, oxaliplatin) polychemotherapies are used for colorectal cancers. Radiotherapy may also be useful, depending on the type of cancer and its location [134].

#### 4.11.2. Fertility Preservation

Chemotherapies used for digestive cancers do not contain alkylating agents and are at low risk of infertility [14]. There is therefore no strict indication for FP when patients receive chemotherapy only. However, if the patient is really demanding, has a good OR, and if time before the start of treatment allows it, it is possible to propose oocyte vitrification after ovarian stimulation. Also, in cases where pelvic radiotherapy is planned, ovarian transposition can be performed [134,135].

## 5. Conclusions

Fertility preservation is a crucial consideration for young women undergoing treatments like cancer treatments that impact fertility. With improved survival rates, focusing on post-treatment quality of life, including reproductive potential, is crucial. Early consultation with reproductive specialists helps determine the best FP strategy based on medical conditions and treatment plans.

Socioeconomic disparities, a lack of awareness among healthcare providers, and time constraints before initiating cancer therapy are all contributors to variations in FP use. It is essential that oncologists are well-informed about available FP options and integrate them into their treatment planning, ensuring that all eligible patients receive appropriate counseling and referral to reproductive specialists. Interdisciplinary collaboration among oncologists and reproductive specialists is critical for addressing the needs of young cancer patients. Integrating FP into standard cancer care allows more women to make informed reproductive choices, enhancing both survival outcomes and life quality after treatment.

Our study provides insights into FP outcomes following gonadotoxic treatments. However, several individual factors, such as age, BMI, smoking status, and prior reproductive history, can influence OR and response to FP strategies. Although these variables were not the primary focus of our analysis, future studies should consider their potential confounding effects to improve FP recommendations in this population.

Future research should refine FP techniques, improve ovarian tissue cryopreservation efficacy, and develop new FP methods such as in vitro follicle growth and maturation. For example, this technique aims to obtain mature metaphase II oocytes from primordial follicles containing diplotene-blocked oocyte I. It involves multi-step in vitro culture systems designed to support both follicular development (from primordial follicle to ovulatory follicle) and oocyte maturation (from oocyte I to mature metaphase II oocyte), including meiotic resumption to achieve full oocyte maturation [136]. Moreover, long-term studies are also needed to evaluate pregnancy rates, offspring health, and the psychological impact of FP on cancer survivors.

Also, post-treatment gynecological follow-up is essential for these patients. Regular monitoring of ovarian function can help detect premature ovarian insufficiency early, allowing gynecologists to properly manage and prevent long-term complications such as osteoporosis, cardiovascular disease, and menopausal symptoms that may impact the quality of life.

INCA recommends that women of childbearing age undergo longitudinal monitoring of AMH before and one year after treatment to assess follicular loss and ovarian toxicity of the different chemotherapy protocols [5]. This monitoring should be conducted at least 12 months post-treatment, as pregnancy is generally not advised until at least 18 months after treatment completion. Also, INCA recommends long-term follow-up to further evaluate the persistence of fertility impairment, spontaneous pregnancy rates, and offspring health.

Finally, FP strategies may be considered post-treatment, particularly for patients experiencing a decline in OR who still wish to conceive in the future. Ensuring access to long-term reproductive care and hormonal management is essential for optimizing fertility outcomes and overall well-being in cancer survivors.

## Figures and Tables

**Table 1:** Gonadotoxicity of common chemotherapy agents based on the risk of treatment-related amenorrhea [6,14].

High Risk of Infertility (>80%)	Intermediate Risk of Infertility (20–80%)	Low Risk of Infertility (<20%)
Alkylating Agents	Platinum Salts Spindle Poisons	Splitting Agents Topoisomerase Inhibitors Antimetabolites
Molecules: cyclophosphamide, ifosfamide, chlorambucil, carmustine, lomustine, melphalan, thiotepa, chlormethine, busulfan, procarbazine	Molecules: cisplatin, carboplatin, paclitaxel, docetaxel, vincristine, vinblastine, adriamycin	Molecules: bleomycin, irinotecan, topotecan, etoposide, epirubicin, methotrexate, 5-fluorouracil, mercaptopurine, hydroxyurea

**Table 2:** Risk of treatment-induced gonadotoxicity in cancer patients associated with the main systemic gonadotoxic therapies [20].

Risk Category	Type of Gonadotoxic Treatment
High risk (>80% risk of treatment-induced amenorrhoea)	Cyclophosphamide-based regimens (with anthracyclines and/or taxanes: (F)EC/(F)AC alone or followed by T or P, TC) in breast cancer patients aged ≥ 40 years Conditioning regimens for HSC transplantation with cyclophosphamide and/or TBI in patients with hematological cancers Abdominal and pelvic radiotherapy to a field that includes the ovaries
Intermediate risk (40–60% risk of treatment-induced amenorrhoea)	Cyclophosphamide-based regimens (with anthracyclines and/or taxanes: (F)EC/(F)AC alone or followed by T or P, TC) in breast cancer patients aged 30–39 years Alkylating agent-based regimens (e.g., MOPP, RSQB, BEACOPP, CHOP, CHOPE) in lymphoma patients
Low risk (<20% risk of treatment-induced amenorrhoea)	Cyclophosphamide-based regimens (with anthracyclines and/or taxanes: (F)EC/(F)AC alone or followed by T or P, TC) in breast cancer patients aged ≤30 years Non-alkylating agent-based regimens (e.g., ABVD or EBVP) in lymphoma patients aged ≥32 years BEP/EP in patients with non-epithelial ovarian cancers FOLFOX, XELOX, or capecitabine in patients with colorectal cancers Multi-agent chemotherapy (EMA-CO and platinum-based combinations) for gestational trophoblastic tumors Radioactive iodine (I-131) in patients with thyroid cancer
Very low or no risk	Targeted agents (trastuzumab, lapatinib, and rituximab)? Tamoxifen and GnRH analog Non-alkylating agent-based regimens (e.g., ABVD or EBVP) in lymphoma patients aged <32 years Single-agent methotrexate
Unknown risk	Platinum- and taxane-based chemotherapy in patients with gynecological and lung cancers Majority of targeted therapies (monoclonal antibodies and small molecules like tyrosine kinase inhibitors) and immunotherapeutic agents

Abbreviations: (F)EC/(F)AC = 5-fluoruracil, epirubicin, doxorubicin, cyclophosphamide; T = docetaxel; P = paclitaxel; GnRH analog = gonadotropin-releasing hormone analog; HSC = hematopoietic stem cell; TBI = total body irradiation; MOPP = mechlorethamine, vincristine, procarbazine, prednisone; RSQB or MOPP/ABV hybrid = MOPP/doxorubicin, bleomycin, vinblastine; BEACOPP = cyclophosphamide, doxorubicin, vincristine, bleomycin, etoposide, procarbazine, prednisone; ABVD = doxorubicin, bleomycin, vinblastine, dacarbazine; EBVP = epirubicin, bleomycin, vinblastine, prednisone; CHOP = cyclophosphamide, doxorubicin, vincristine, prednisone; CHOPE = CHOP plus etoposide; BEP = etoposide, cisplatin, bleomycine; EP = etoposide, cisplatin; FOLFOX = 5-fluoruracil, oxaliplatin; XELOX = capecitabine, oxaliplatin; EMA-CO = etoposide, actinomycinD, methotrexate followed by cyclophosphamide and vincristine.
